# The SIRT6 activator MDL‐800 improves genomic stability and pluripotency of old murine‐derived iPS cells

**DOI:** 10.1111/acel.13185

**Published:** 2020-07-21

**Authors:** Yu Chen, Jiayu Chen, Xiaoxiang Sun, Jiayu Yu, Zhen Qian, Li Wu, Xiaojun Xu, Xiaoping Wan, Ying Jiang, Jian Zhang, Shaorong Gao, Zhiyong Mao

**Affiliations:** ^1^ Clinical and Translational Research Center of Shanghai First Maternity & Infant Hospital, Shanghai Key Laboratory of Signaling and Disease Research, Frontier Science Center for Stem Cell Research, School of Life Sciences and Technology Tongji University Shanghai China; ^2^ Tsingdao Advanced Research Institute Tongji University Qingdao China; ^3^ State Key Laboratory of Natural Medicines China Pharmaceutical University Nanjing China; ^4^ Department of Pathophysiology, Key Laboratory of Cell Differentiation and Apoptosis of Ministry of Education Shanghai Jiao‐Tong University School of Medicine Shanghai China

**Keywords:** aging, DNA repair, genome integrity, MDL‐800, pluripotency, SIRT6

## Abstract

Cellular reprogramming is an emerging strategy for delaying the aging processes. However, a number of challenges, including the impaired genome integrity and decreased pluripotency of induced pluripotent stem cells (iPSCs) derived from old donors, may hinder their potential clinical applications. The longevity gene, Sirtuin 6 (SIRT6), functions in multiple biological processes such as the maintenance of genome integrity and the regulation of somatic cell reprogramming. Here, for the first time, we demonstrate that MDL‐800, a recently developed selective SIRT6 activator, improved genomic stability by activating two DNA repair pathways—nonhomologous end joining (NHEJ) and base excision repair (BER) in old murine‐derived iPSCs. More interestingly, we found that pretreating old murine iPSCs, which normally exhibit a restricted differentiation potential, with MDL‐800 promoted the formation of teratomas comprised of all three germ layers and robustly stimulated chimera generation. Our findings suggest that pharmacological activation of SIRT6 holds great promise in treating aging‐associated diseases with iPSC‐based cell therapy.

## Graphical abstract

MDL‐800, a selective SIRT6 activator, improves genomic stability by activating nonhomologous end joining and base excision repair, and enhances the pluripotency of old murine‐derived iPSCs.

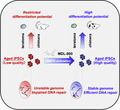

## 1 INTRODUCTION, RESULTS, AND DISCUSSION

Aging is a complex process characterized by a time‐dependent decline in physiological function and an increased vulnerability to disease (Chalkiadaki & Guarente, 2015; Kennedy et al., 2014). The loss of tissue homeostasis contributes to the onset of aging and age‐related diseases (Sullivan et al., 2018). Restoring the functionality of aged tissues with adult stem cells is an emerging strategy in regenerative medicine (Cerletti et al., 2008; Chhabra & Brayman, 2013). For instance, hematopoietic progenitor cell transplantation is an FDA‐approved treatment for reconstituting the hematopoietic and immunologic systems in patients. In addition, several other types of stem cell therapies are under investigation in clinical trials to evaluate their safety and effectiveness in treating diseases, including type 2 diabetes mellitus, Parkinson's disease, and osteoarthritis (https://clinicaltrials.gov). However, only a limited number of types of adult stem cells can be successfully isolated, maintained *in vitro*, and applied in the clinic (Mount, Ward, Kefalas, & Hyllner, 2015). Induced pluripotent stem cells (iPSCs) derived from autologous somatic cells transduced with Yamanaka factors can be subsequently differentiated into desired cell types *in vitro* and further applied to treat age‐related diseases in a variety of types of tissues (Park et al., 2008; Si‐Tayeb et al., 2010; Takebe et al., 2013). Additionally, autologous therapy with iPSCs may avoid certain ethical concerns and potentially minimize the risk of immune rejection associated with allogenic stem cell products. However, the potential clinical application of iPSCs in regenerative medicine faces a dilemma as it is likely that the iPSCs used to treat any age‐related diseases would be derived from somatic cells isolated from patients at old ages while the quality of iPSCs derived from old donors is not as high as young iPSCs or embryonic stem cells (ESCs) (Lo Sardo & Ferguson, 2017; Skamagki et al., 2017). Genome integrity and pluripotent potential are two critical parameters in evaluating the quality of iPSCs (Sullivan et al., 2018). Developing novel methods to improve the genome integrity and pluripotency of iPSCs derived from old subjects would help achieve therapeutic goals in treating age‐related diseases.

The longevity gene SIRT6 is an enzyme possessing both NAD^+^‐dependent protein deacetylase activity and mono (ADP‐ribosyl) transferase activity. Loss of SIRT6 leads to genomic instability and severe phenotypes consistent with premature aging, including osteopenia, reduced subcutaneous fat, and shortened life span in mice (Mostoslavsky et al., 2006), while overexpression of SIRT6 extends murine life span (Kanfi et al., 2012). Several reports demonstrate that SIRT6 is a pivotal regulator of different DNA repair pathways, including the DNA double‐strand break (DSB) repair pathways—canonical nonhomologous end joining (c‐NHEJ), alternative NHEJ (alt‐NHEJ), homologous recombination (HR)—and base excision repair (BER) by targeting DNA‐PKcs, PARP1 and SNF2H (Mao et al., 2011, 2012; McCord et al., 2009; Toiber et al., 2013; Xu et al., 2015). As a chromatin associated epigenetic factor, SIRT6 also participates in regulating the expression of the pluripotency genes that determine the balance between pluripotency and differentiation (Etchegaray et al., 2015; O'Callaghan & Vassilopoulos, 2017). Although expression of SIRT6 protein gradually increases during reprogramming (Xu et al., 2019), our previous study found that SIRT6 protein level is significantly lower in old murine‐derived iPSCs and that the low expression of SIRT6 resulted in the decline of NHEJ and genomic stability in old murine‐derived iPSCs compared to those derived from young mice (Chen et al., 2017). However, whether SIRT6 activators can be utilized to enhance DNA repair to stabilize genomes and improve the pluripotency of old iPSCs remains to be determined.

**FIGURE 1:**
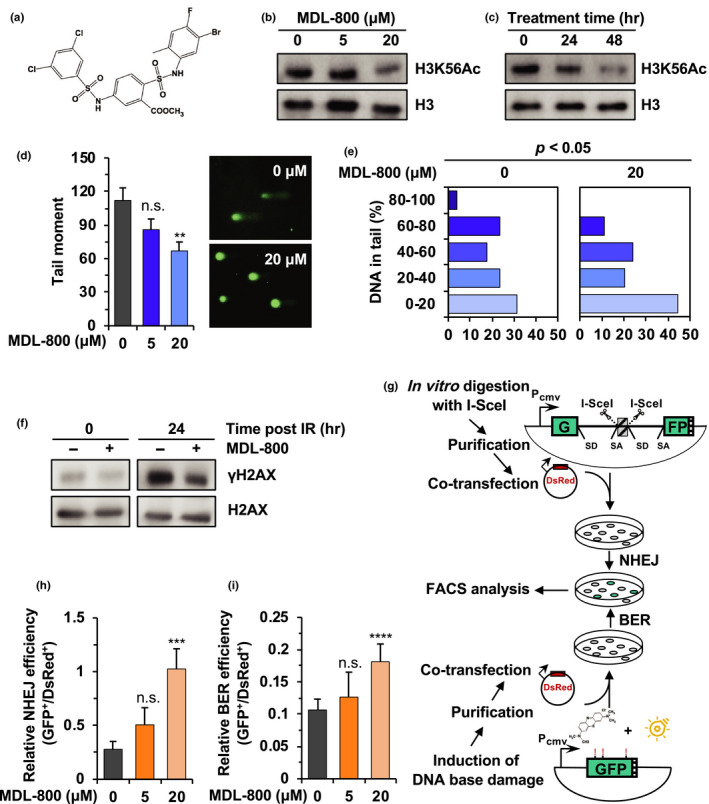
MDL‐800 promotes genome integrity by enhancing NHEJ and BER in old murine‐derived iPSCs. (a) Chemical structure of MDL‐800. (b) H3K56Ac levels in old murine‐derived iPSCs treated with the indicated doses of MDL‐800 for 24 hr. (c) H3K56Ac levels in old murine‐derived iPSCs treated with 20 μΜ MDL‐800 for indicated time period. (d‐e) Analysis of genome integrity in old murine‐derived iPSCs treated with the indicated doses of MDL‐800 for 5 passages by alkaline comet assay. Data in (d) and (e) are from the same experiment. The average tail moments are shown in (d), and the percentage of DNA content in tails are shown in (e). At least 50 cells per group were included for analysis. Error bars represent *SEM*. *p* value in (e) was determined by ANOVA. (f) Western blot analysis of γH2AX level. Old murine‐derived iPSCs were treated with 20 μΜ MDL‐800 for 5 passages before X‐ray irradiation at 8 Gy. Cells were lysed for protein extraction at the indicated time point post‐X‐ray treatment. (g) The schematic depictions of NHEJ and BER efficiency assay. For NHEJ efficiency analysis, the NHEJ reporter was linearized by I‐SceI endonuclease *in vitro* to mimic DSBs. For BER efficiency analysis, the pEGFP‐N1 plasmid was mixed with methylene blue and exposed to visible light produced by a 100‐W bulb for 120 min to induce base damage. The purified linearized NHEJ reporter (0.4 μg) or damaged pEGFP‐N1 reporter (0.2 μg), along with 0.1 μg pCAG‐DsRed vector, was transfected into 2 × 10^5^ mouse iPSCs. FACS analysis was performed at 48‐hr post‐transfection. (h‐i) Analysis of NHEJ and BER efficiency of old murine‐derived iPSCs treated with indicated doses of MDL‐800 for 5 passages. Error bars represent s.d.. All experiments were repeated at least three times. **p < 0.01, ***p < 0.001, ****p < 0.0001, n.s. not significant

MDL‐800 (Figure 1a) is a selective allosteric activator of SIRT6. It stimulates SIRT6 catalytic activity and promotes the binding affinities of substrate to SIRT6 (Huang et al., 2018). We therefore tested whether MDL‐800 treatment improved the quality of old murine (2‐year‐old)‐derived iPSCs. We first validated that MDL‐800 enhances the enzymatic activity of mouse SIRT6. We pretreated the iPSCs derived from the old mice with MDL‐800 at concentrations of 5 μM and 20 μM for 24 hr, and then analyzed the level of a SIRT6 substrate H3K56Ac. We found that, consistent with previous reports on human SIRT6, treating the mouse iPSCs with MDL‐800 at 20 μM promoted the deacetylation of H3K56Ac (Figure 1b), and prolonging the incubation time to 48 hr led to a further reduction in acetylation level of H3K56Ac (Figure 1c). These data indicate that MDL‐800 might also directly activate the catalytic activity of mouse SIRT6. To further demonstrate that MDL‐800 affects the acetylation level of H3K56Ac through monitoring mouse SIRT6 activity, we treated iPSCs derived from *Sirt6* ^+/+^ mouse embryonic fibroblasts (MEFs) or *Sirt6* ^−/−^ MEFs with MDL‐800. We found that *Sirt6* deficiency abrogated the MDL‐800 mediated promotion of H3K56Ac deacetylation (Figure S1a,b). Taken together, our results indicate that MDL‐800 specifically activates SIRT6 enzymatic activity in mouse iPSCs.

To evaluate the effects of MDL‐800 on genomic stability in iPSCs derived from old mice, we performed alkaline comet assays. We observed a dosage‐dependent decline in tail moment, which reflects genomic instability, in MDL‐800 treated iPSCs derived from old mice (Figure 1d). A similar result assayed by the percentage of DNA content in the tail was also obtained (Figure 1e). Moreover, we also examined genomic stability using comet assays in two additional clones of old murine‐derived iPSCs (Figure S2a,b), and we observed a nearly identical decrease in tail moment, ruling out the possibility that the function of MDL‐800 is clone‐specific. Consistent with these observations, γH2AX (S139) level, a classical marker of DNA damage, was also reduced in old murine‐derived iPSCs treated with MDL‐800 in the absence or presence of X‐ray irradiation (Figure 1f). These results suggest that treating old murine‐derived iPSCs with MDL‐800 is an effective method to stabilize the genome.

Efficient DNA repair is critical to the maintenance of genome integrity. Defects in different types of DNA repair pathways such as c‐NHEJ or BER often lead to phenotypes of premature aging (Li et al., 2018; Lombard et al., 2005). We hypothesized that MDL‐800 promotes genomic stability by boosting DNA repair. Considering SIRT6 participates in repairing both DSBs and DNA damage at bases, we set out to investigate which pathway could be activated post‐MDL‐800 treatment using our previously reported extrachromosomal repair assay (Seluanov, Mao, & Gorbunova, 2010; Zhang et al., 2020). The GFP‐based NHEJ or HR cassettes were linearized by I‐SceI endonuclease *in vitro* to mimic DSBs. Then, the linearized NHEJ (0.4 μg) or HR (0.5 μg) reporter, along with 0.1 μg pCAG‐DsRed vector for monitoring the difference in transfection efficiency between experiments, was transfected into 2 × 10^5^ mouse iPSCs. FACS analysis was performed at 48‐hr post‐transfection (Figure 1g and Figure S3a). We found that 20 μM MDL‐800 treatment significantly promoted NHEJ efficiency by 4‐fold, while it did not influence HR repair (Figure 1h and Figure S3b). Treatment with a lower dosage (5 μM) also showed a trend of NHEJ enhancement, although the difference was not statistically significant (Figure 1h). This result is consistent with our previous study which demonstrated that different from its function in somatic cells, SIRT6 regulates NHEJ rather than HR in mouse iPSCs (Chen et al., 2017), suggesting a cell type‐specific role for SIRT6 in regulating DSB repair. The efficiency of DSB repair was also analyzed in iPSCs generated from *Sirt6* ^+/+^ and *Sirt6* ^−/−^ MEFs (Figure S5a,b), and our data demonstrated that MDL‐800 activated NHEJ in a SIRT6‐dependent manner. Previous studies have indicated that the choice of DSB repair pathways is determined by cell cycle stage (Hustedt & Durocher, 2016; Mao, Bozzella, Seluanov, & Gorbunova, 2008). To rule out the possibility that the MDL‐800‐mediated stimulatory effect on NHEJ is dependent on cell cycle arrest, we performed EdU incorporation assays (Figure S4). We did not find any difference in cell cycle distribution between control and 20 μM MDL‐800‐treated old murine‐derived iPSCs (Figure S4), suggesting that the SIRT6 activator MDL‐800 promotes NHEJ in old murine‐derived iPSCs in a cell cycle‐independent manner.

Moreover, SIRT6 was reported to regulate BER in both mice and humans (Mostoslavsky et al., 2006; Xu et al., 2015). We then investigated whether MDL‐800 treatment also influences the BER pathway using our previously established plasmid reactivation assay (Xu et al., 2015; Zhang et al., 2020). Briefly, 10 μg pEGFP‐N1 plasmid was mixed with methylene blue, followed by a 120‐min exposure to visible light generated by a 100‐W bulb, which induces 8‐hydroxyguanine damage on plasmids. Then, 0.2 μg purified damaged pEGFP‐N1 plasmid, together with 0.1 μg pCAG‐DsRed vector for normalizing transfection efficiency, was transfected into 2 × 10^5^ mouse iPSCs, followed by FACS analysis at 48‐hr post‐transfection (Figure 1g). We found that the efficiency of BER also showed a 2‐fold increase post‐20 μM MDL‐800 treatment in old murine‐derived iPSCs (Figure 1i). Similarly, the MDL‐800‐mediated stimulatory effect on BER efficiency was only observed in *Sirt6* ^+/+^ mouse iPSCs, but not in *Sirt6* ^−/−^ mouse iPSCs, which further validated that MDL‐800 functions through activating SIRT6 (Figure S5c).

Taken together, these data reveal that in a SIRT6‐dependent fashion, MDL‐800 stimulates both NHEJ and BER in iPSCs derived from old mice, therefore stabilizing genomes of these iPSCs.

**FIGURE 2:**
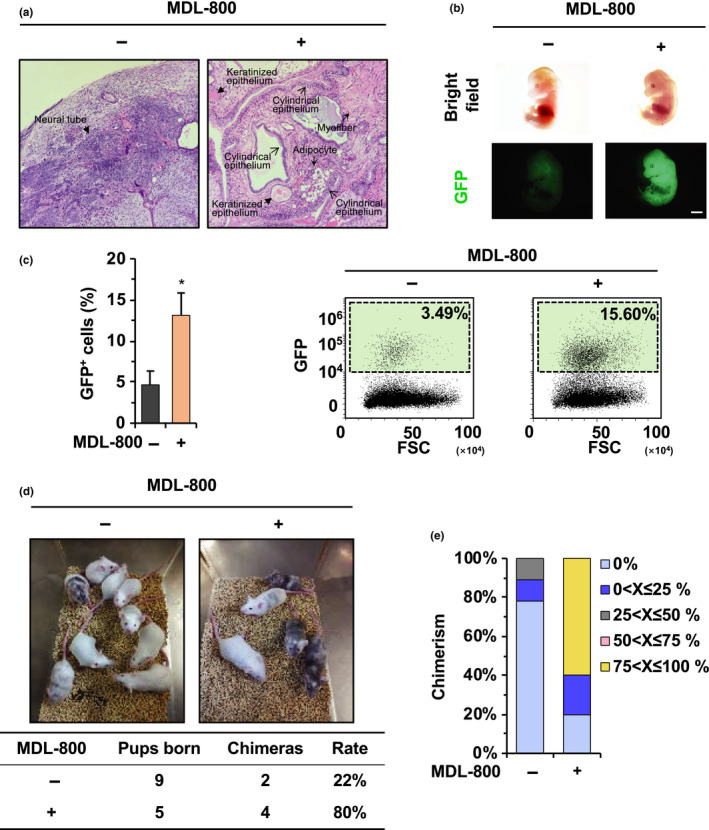
MDL‐800 improves the differentiation potential of old murine‐derived iPSCs. (a) MDL‐800 promotes the formation of teratomas comprised of all three germ layers from old murine‐derived iPSCs while teratomas in the control group show a neuroectoderm‐skewed differentiation phenotype. Old murine iPSCs were treated with 20 μΜ MDL‐800 for 5 passages before injection. Teratomas were dissected for HE staining. Ectoderm: neural tube, keratinized epithelium; Mesoderm: myofiber, adipocyte; Endoderm: cylindrical epithelium. (b‐c) MDL‐800 promotes chimera formation from GFP‐tagged old murine‐derived iPSCs. GFP‐tagged old murine‐derived iPSCs were treated with 20 μΜ MDL‐800 for 5 passages before blastocyst microinjection. Representative fluorescent images of E14.5 chimeric mouse embryos are shown in (b). Scale bar: 2 mm. The percentage of GFP positive cells in E14.5 embryos (left panel) and the representative FACS traces (right panel) are shown in (c). (d‐e) MDL‐800 promotes the generation of mice with higher chimerism from old murine‐derived iPSCs. iPSCs were treated with 20 μΜ MDL‐800 for 5 passages before blastocyst microinjection. Representative images of adult chimeric mice (upper panel) and the rate of chimera formation (lower panel) are shown in (d). The degree of chimerism was evaluated by the coat color in (e). Error bars represent *SEM*, *p < 0.05

In addition to genome integrity, the pluripotency of iPSCs is another critical quality attribute (Sullivan et al., 2018). Previous studies have reported that SIRT6 participates in the regulation of pluripotency in both iPSCs and ESCs (Etchegaray et al., 2015; Xu et al., 2019). Thus, we set out to test whether MDL‐800 treatment could positively regulate the pluripotency and differentiation potential of old murine‐derived iPSCs. Mouse iPSCs pretreated with or without MDL‐800 were injected subcutaneously into the groin of immunodeficient nude mice. One month after injection, teratomas were dissected for further analysis. Hematoxylin–eosin (HE) staining demonstrated that MDL‐800 treated mouse iPSCs supported the formation of teratomas with all three germ layers, whereas untreated cells showed a neuroectoderm‐skewed differentiation phenotype (Figure 2a and Figure S6), which was similar to that of *Sirt6* ^−/−^ ESCs (Etchegaray et al., 2015). Moreover, the pluripotency and *in vivo* differentiation potential of mouse iPSCs were evaluated by chimera experiments. Old C57BL/6 murine‐derived iPSCs were first infected with lentivirus bearing vectors encoding GFP for labeling, and the GFP^+^ cells were sorted by FACS. GFP^+^ mouse iPSCs were pretreated with MDL‐800 before microinjection into blastocysts. Then, the embryos were transplanted into the uteruses of pseudo‐pregnant ICR mice. E14.5 chimeric embryos were obtained by cesarean sections and used for chimerism analysis. Strikingly, we found that embryos generated from mouse iPSCs treated with MDL‐800 showed a stronger green fluorescence, indicating a higher capacity to differentiate into three lineages in chimeric mice (Figure 2b). In addition, the skins of embryos were dissociated into single cells and assessed by FACS for quantitative analysis. The result clearly showed that the percentage of GFP^+^ cells were approximately 3‐fold higher in MDL‐800 treated group than in the control group (Figure 2c), indicating an improvement in *in vivo* differentiation of the same cell line upon MDL‐800 treatment. Further evidence from agouti coat color of the adult mice gave a similar result. There was a remarkable increase in the percentage of mice with a chimeric black coat color in the MDL‐800 treated group as compared to control group (Figure 2d,e).

To further validate that MDL‐800 promotes pluripotency in old murine‐derived iPSCs in a SIRT6‐dependent manner, *Sirt6* ^+/+^ and *Sirt6* ^−/−^ mouse iPSCs were also labeled in green fluorescence for chimera analysis. Embryos post‐transplantation at E14.5 were obtained for imaging under a fluorescent microscope (Figure S7a) and were further digested for analyzing the percentage of GFP^+^ cells by FACS (Figure S7b). We found that chimera formation was significantly promoted by 2.4‐fold from *Sirt6* ^+/+^ mouse iPSCs but not from *Sirt6* ^−/−^ mouse iPSCs upon MDL‐800 treatment (Figure S7b). Collectively, these data demonstrated that MDL‐800 promotes pluripotency of old murine‐derived iPSCs in a SIRT6‐dependent manner.

Cumulatively, we demonstrate that activating SIRT6 with the recently developed potent SIRT6 activator, MDL‐800, improves the quality of iPSCs derived from old mice. It activates different pathways of DNA repair including NHEJ and BER, thereby promoting genome integrity; and it also improves the differentiation potential of old murine‐derived iPSCs. Our data imply that the safe and controlled pharmaceutical activation of SIRT6 with MDL‐800 holds great potentials in iPSC‐based cell therapy in treating aging‐associated diseases. Nevertheless, whether it has similar functions in iPSCs derived from old patient cells needs to be further determined, although a number of reports have indicated that the age‐associated decline in SIRT6 expression is possibly the determining factor causing the rise in genomic instability in human cells (Rohani, Johnson, Arnold, & Stolzing, 2014; Sharma et al., 2013; Xu et al., 2015).

## CONFLICT OF INTEREST

None declared.

## AUTHOR CONTRIBUTIONS

Y.C., J.C., and X.S. performed experiments, analyzed data, and wrote manuscript. J.Y., Z.Q., L.W., X.X., X.W., and Y.J. were involved in collection of data. J.Z., S.G., and Z.M. were involved in the conception and design, data interpretation, manuscript writing, and final approval of manuscript.

## Supporting information

Fig S1‐S7

Supplementary Material

## Data Availability

Data sharing is not applicable to this article as no new data were created or analyzed in this study.
