# Supplementary material for: The SIRT6 activator MDL‐800 improves genomic stability and pluripotency of old murine‐derived iPS cells
Source: Aging Cell. 2020 Jul 21;19(8):e13185. doi: 10.1111/acel.13185 (PMC7431819; doi:10.1111/acel.13185)
Supplement: Supplementary file 1 — Fig S1‐S7 [file ACEL-19-e13185-s001.pdf]

(a)

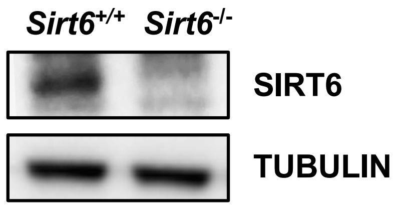


(b)

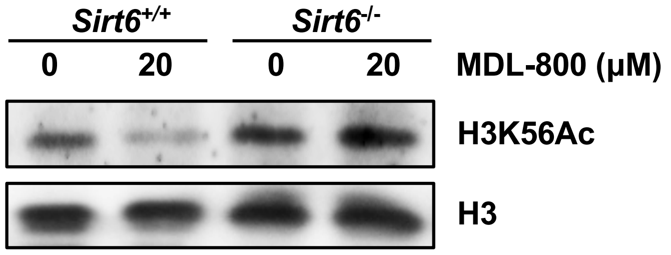


# Supplementary figure 1

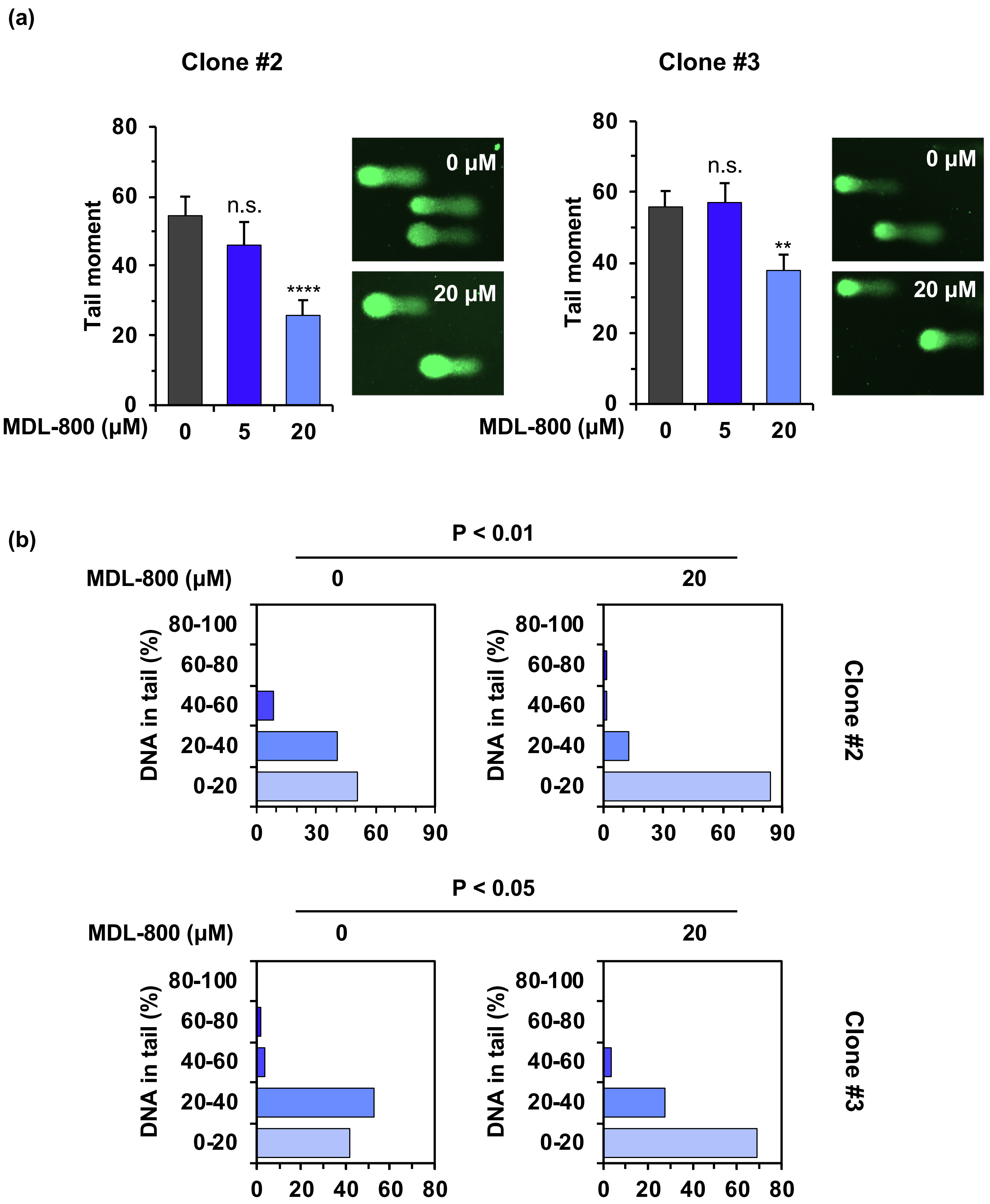


# Supplementary figure 2

(a)

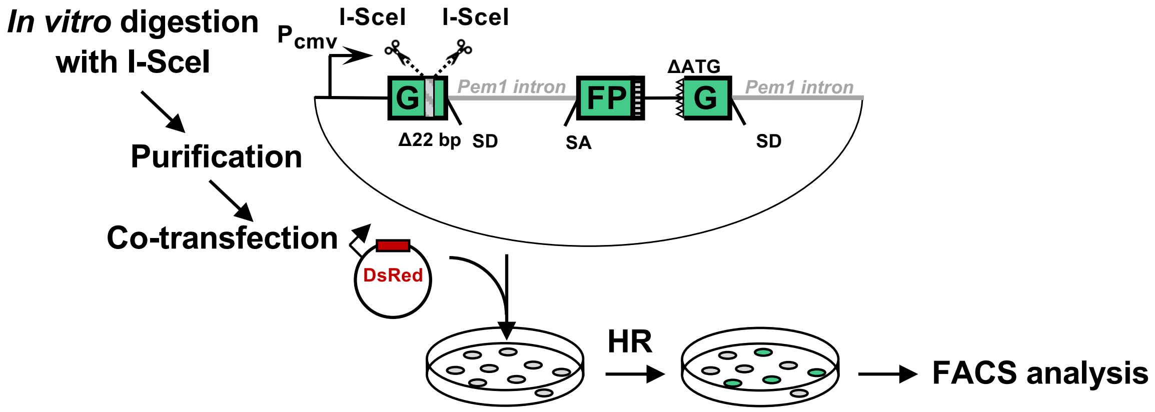


(b)

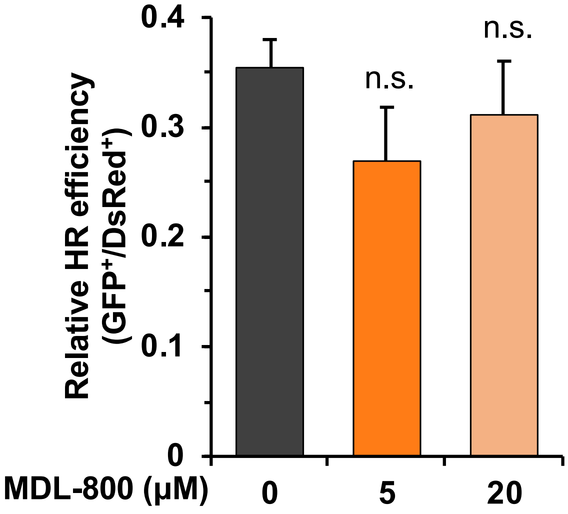


# Supplementary figure 3

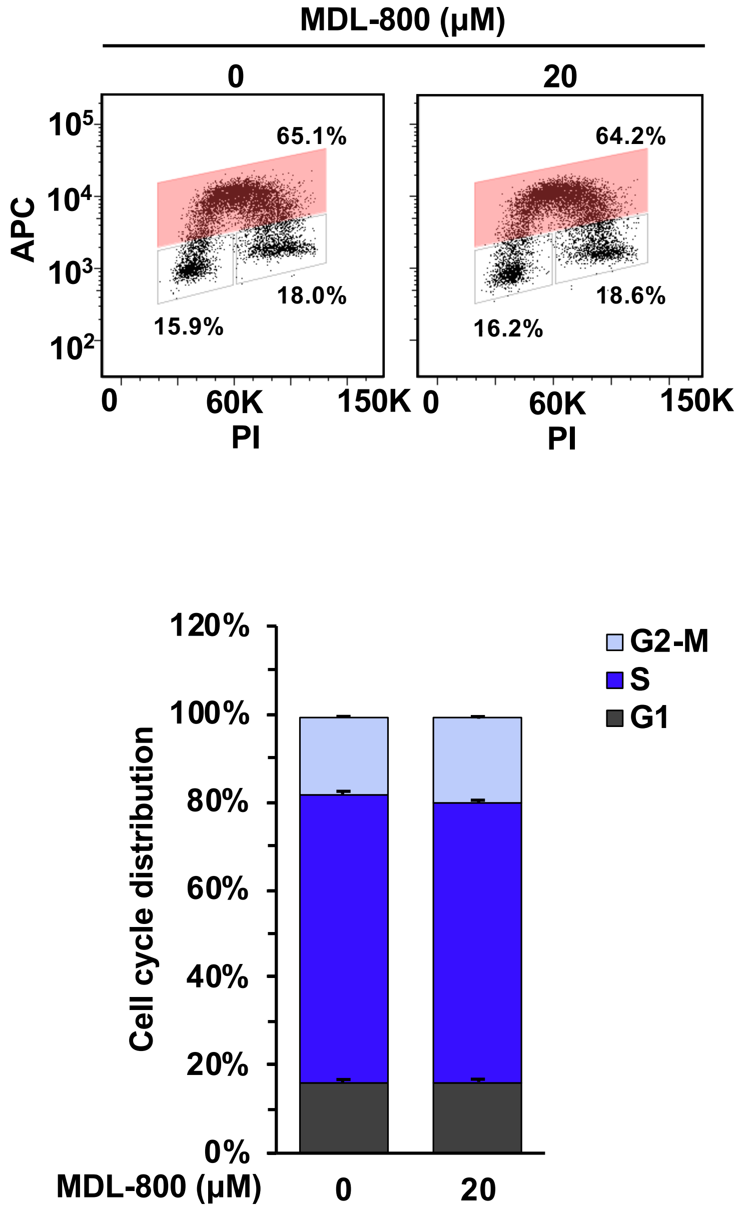


# Supplementary figure 4

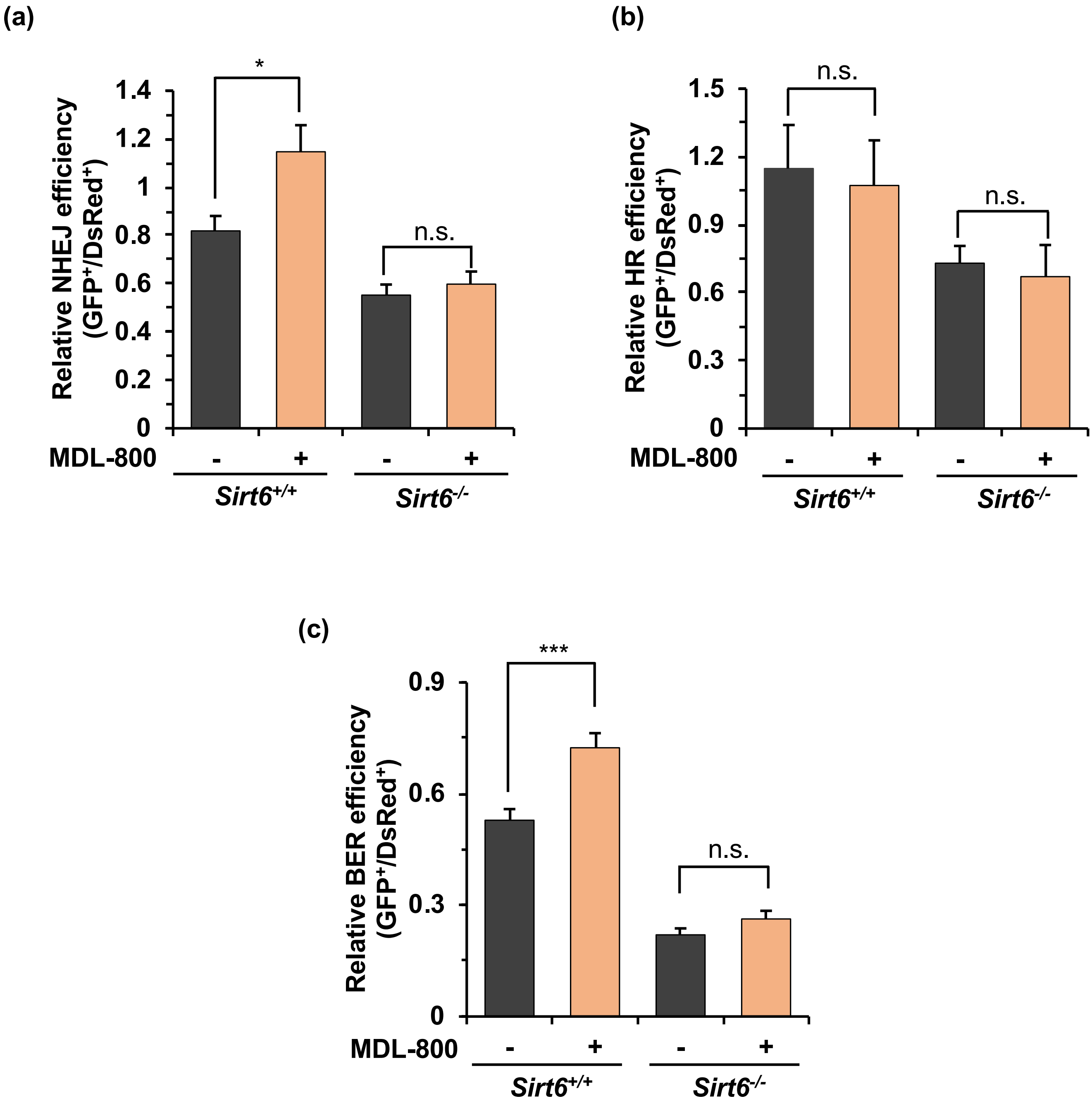


# Supplementary figure 5

(a)

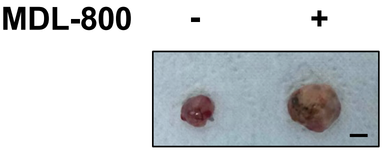


(b)

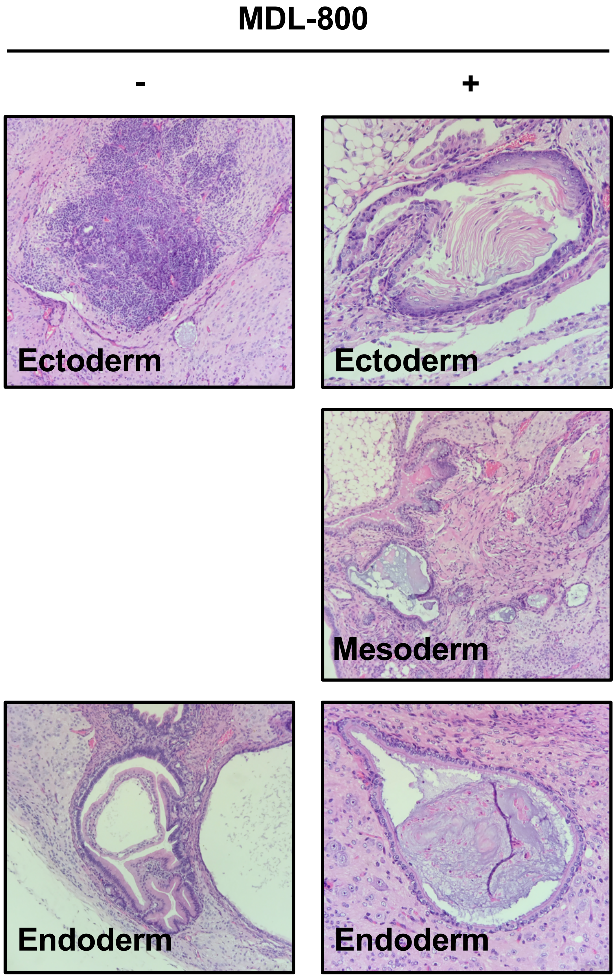


# Supplementary figure 6

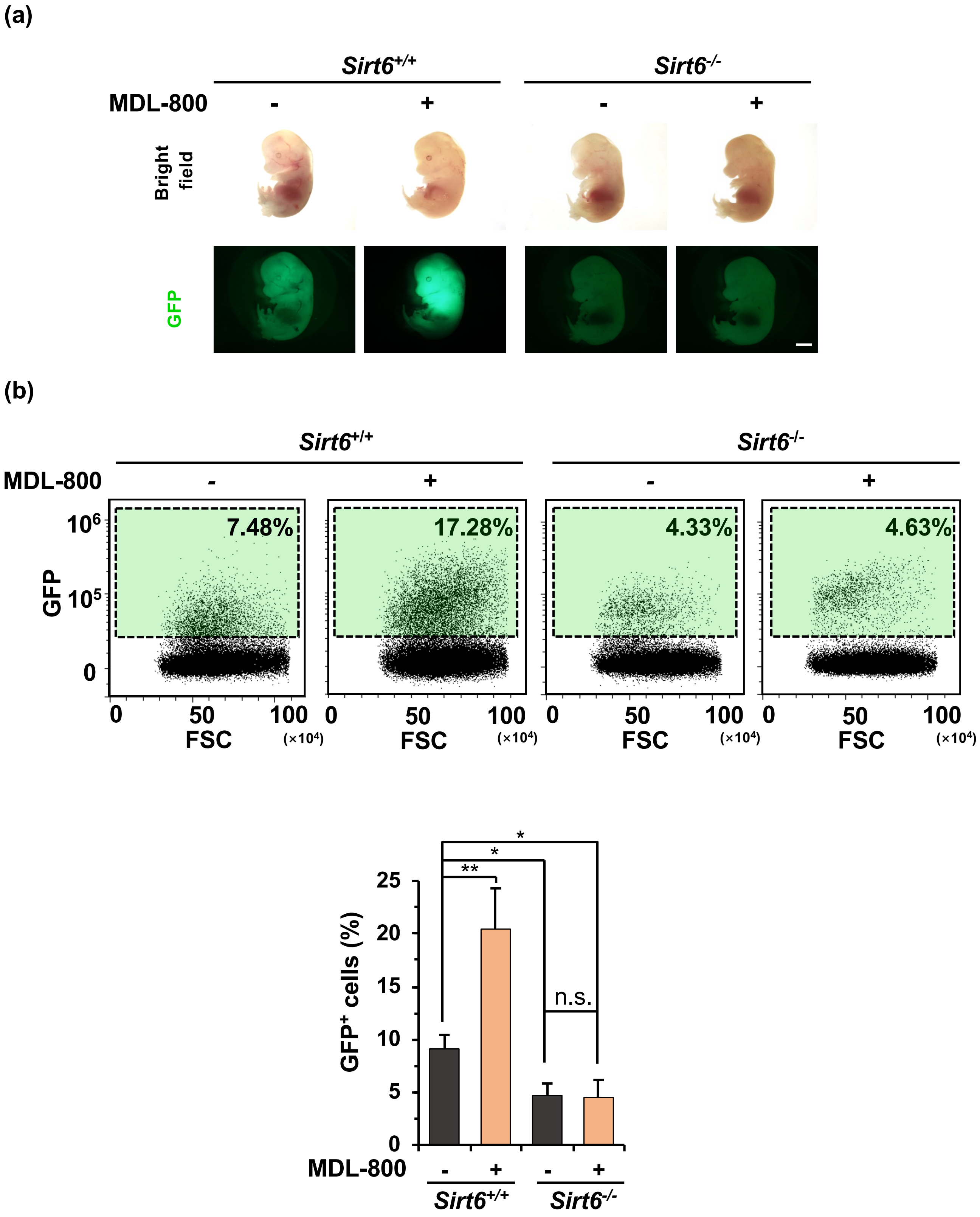


# Supplementary figure 7
