# Supplementary material for: The SIRT6 activator MDL‐800 improves genomic stability and pluripotency of old murine‐derived iPS cells
Source: Aging Cell. 2020 Jul 21;19(8):e13185. doi: 10.1111/acel.13185 (PMC7431819; doi:10.1111/acel.13185)
Supplement: Supplementary file 2 — Supplementary Material [file ACEL-19-e13185-s002.docx]

## SUPPORTING INFORMATION

- **Experimental procedures**
- **Supplementary Figures (Figure S1-S7)**

**EXPERIMENTAL PROCEDURES**

**Animal use and care**

The specific pathogen-free (SPF) grade mice, including ICR and immunodeficient nude mice, were housed in the animal facility at Tongji University, Shanghai, China. All the mice had free access to food and water. All the experiments were performed in accordance with the University of Health Guide for the Care and Use of Laboratory Animals and were approved by the Biological Research Ethics Committee of Tongji University.

**Cell culture and treatment**

The old murine-derived iPSCs used in this manuscript were from our previous study ([W. Chen et al., 2017](#_ENREF_2)). These iPSCs were induced from skin fibroblasts derived from the chest and belly of two-year-old wild-type C57BL/6 mice following a previously reported protocol ([Okita, Ichisaka, & Yamanaka, 2007](#_ENREF_3)). Mouse iPSCs were cultured on mitomycin C-treated mouse embryonic fibroblasts in ES medium containing DMEM (Corning Incorporated, Corning, USA, Cat. # 10-013-CVR) supplemented with 15% fetal bovine serum (Gibco, Carlsbad, USA, Cat. # 16000-044), 1 × nonessential amino acid (Gibco, Carlsbad, USA, Cat. # 11140-050), 1% penicillin/streptomycin (Gibco, Carlsbad, USA, Cat. # 15140-122), 1 mM L-glutamine (Gibco, Carlsbad, USA, Cat. # 35050-061), 0.1 mM 2-mercaptoethanol (Millipore, Darmstadt, Germany, Cat. # ES-008-D), 1 × nucleosides (Millipore, Darmstadt, Germany, Cat. # ES-007-E), 1,000 U/mL LIF (Millipore, Darmstadt, Germany, Cat. # ESG1107), 1 μM PD0325901 (Selleckchem, Houston, USA, Cat. # S1036) and 3 μM CHIR-99021 (Selleckchem, Houston, USA, Cat. # S2924). HEK293FT cells were cultured in DMEM (Corning Incorporated, Corning, USA, Cat. # 10-013-CVR) supplemented with 10% fetal bovine serum (Gibco, Carlsbad, USA, Cat. # 16000-044) and 1% penicillin/streptomycin (Gibco, Carlsbad, USA, Cat. # 15140-122). Cells were maintained in a 5% CO_2_ and 3% O_2_ humidified incubator (Thermo Fisher Heracell 240i) at 37 °C. iPSCs were treated with 20 μM MDL-800 for 5 passages (unless otherwise noted) before analysis of DNA repair efficiency or pluripotency.

**Antibodies**

The antibodies used in this study are as follows: H3 (Cell signaling, Cat. # 9715S), Acetyl-Histone H3 (Lys56) (Invitrogen, Cat. # PA5-40101), H2AX (Abcam, Cat. # ab20669), γH2AX (S139) (Cell signaling, Cat. # 9718S), SIRT6 (Abacm, Cat. # ab62739), β-TUBULIN (Bioworld, Cat. # AP0064).

**Analysis of DNA repair efficiency**

For analysis of HR and NHEJ efficiency, an I-SceI linearized NHEJ (0.4 μg) or HR (0.5 μg) cassette, together with a pCAG-DsRed vector (0.1 μg) were electroporated into 2×10^5^ mouse iPSCs with the CG104 program on a Lonza 4D machine (Lonza, Cologne, Germany). For BER efficiency analysis, a pEGFP-N1 vector treated with methylene blue plus vision light (0.2 μg) was co-transfected with a pCAG-DsRed vector (0.1 μg) into 2×10^5^ mouse iPSCs with the CG104 program. 48 h post transfection, cells were harvested for FACS analysis on FACSverse (BD Biosciences). The FACS data were analyzed by FlowJo (Ashland, OR, USA), and DNA repair efficiency was calculated as the ratio of GFP^+^/DsRed^+^ cells.

**Comet assay**

Mouse iPSCs were resuspended in 1 × PBS to a concentration of 3 × 10^5^ cells/mL for alkaline comet assay using a comet assay kit (Trevigen, Cat. # 4250-050-K). The cells were mixed with molten LMAgarose at a ratio of 1:10 (v/v) and pipetted onto the slides. The slides were then placed at 4 °C for 30 minutes followed by 1-hour lysis and 1-hour unwinding. Then alkaline electrophoresis was performed with the CometAssay ES unit at 21 volts for 30 minutes. The slides were immersed in dH_2_O twice and in 70% ethanol once for 5 minutes each before being dried at 37 °C and stained with SYBR green at 4 °C. The tail moments and the percentage of DNA content in tails was analyzed using Cometscore software. At least 50 cells per group were included for comet analysis.

**EdU incorporation assay**

Cells were treated with 20 μM MDL-800 for 5 passages before EdU incorporation assay. The cells were incubated with 10 μM EdU in a 5% CO_2_ and 3% O_2_ humidified incubator (Thermo Fisher Heracell 240i) at 37 °C for 2 hours. Then cells were harvested for fixation, permeabilization and the Click-iT reaction. Propidium iodide was used for DNA content staining post RNase treatment. The detailed procedures were as described in the protocols provided in Click-iT EdU Assay Kit (Invitrogen, Cat. # C10634). FACS analysis was performed on FACSverse (BD Biosciences) and the FACS data were analyzed by FlowJo (Ashland, OR, USA).

**Lentivirus packaging and infection**

At 24 h post seeding, HEK293FT cells were transfected with 2.5 μg VSVG, 3.75 μg delta 8.9 and 5 μg Lentivirus-EGFP using p-PEI reagent. At 72h post transfection, the culture medium was filtered and concentrated with PEG8000 at 4 °C overnight. Then virus particles were collected after centrifugation at 5000 rpm at 4 °C for 30 minutes, and the virus pellets were resuspended with ES medium. The medium containing virus particles was added to the culture of iPSCs for infection. After 8-hour incubation at 37 °C, the medium was replaced with fresh ES medium. GFP^+^ cells were sorted with Moflo Astrios (Beckman Coulter).

**Teratomas**

The detailed procedures were described previously ([J. Chen et al., 2015](#_ENREF_1)). Briefly, old murine-derived iPSCs (5 × 10^5^) with or without 5-passage treatment of MDL-800 were subcutaneously injected into groins of immunodeficient nude mice. Four weeks after injection, the teratomas were dissected. The teratomas were then fixed in 4% formaldehyde and sent to Shanghai Jeayea Biotech for hematoxylin-eosin (HE) staining.

**Chimeras**

To produce chimeric mice, 10-15 mouse iPSCs with a 5-passage MDL-800 pre-treatment were microinjected into ICR blastocysts using a piezo-actuated microinjection pipette. The embryos were then transplanted into the uteruses of pseudo-pregnant ICR mice. For chimeras generated from GFP tagged mouse iPSCs, E14.5 embryos were obtained by caesarean sections for imaging and were further dissociated for analyzing percentage of GFP^+^ cells on CytoFLEX LX (Beckman). For chimeras generated from non-GFP-tagged mouse iPSCs, the degree of chimerism were evaluated by the coat color of progeny.

**REFERENCE**

Chen, J., Gao, Y., Huang, H., Xu, K., Chen, X., Jiang, Y., . . . Gao, S. (2015). The combination of Tet1 with Oct4 generates high-quality mouse-induced pluripotent stem cells. *Stem Cells, 33*(3), 686-698. doi: 10.1002/stem.1879

Chen, W., Liu, N., Zhang, H., Zhang, H., Qiao, J., Jia, W., . . . Kang, J. (2017). Sirt6 Promotes DNA End Joining in iPSCs Derived from Old Mice. *Cell Rep, 18*(12), 2880-2892. doi: 10.1016/j.celrep.2017.02.082

Okita, K., Ichisaka, T., & Yamanaka, S. (2007). Generation of germline-competent induced pluripotent stem cells. *Nature, 448*(7151), 313-317. doi: 10.1038/nature05934

**SUPPLEMENTARY FIGURE LEGENDS**

**Supplementary figure 1. MDL-800 promotes deacetylation of H3K56Ac in a *Sirt6*-dependent manner.** (a) Western blot analysis of SIRT6 in *Sirt6^-/-^* iPSCs. (b) MDL-800 promotes deacetylation of H3K56Ac in *Sirt6*^+/+^ but not in *Sirt6*^-/-^ mouse iPSCs.

**Supplementary figure 2. MDL-800 stabilizes the genome of old murine-derived iPSCs.** Analysis of genome integrity of two additional clones of old murine-derived iPSCs treated with the indicated doses of MDL-800 for 5 passages by alkaline comet assay. For each clone, data in (a) and (b) are from the same experiment. The average tail moments are shown in (a) and the percentage of DNA content in tails are shown in (b). At least 50 cells per group were included for analysis. Error bars represent s.e.m., ** *P* < 0.01, **** *P* < 0.0001, n.s. not significant. *P* value in (b) was determined from ANOVA.

**Supplementary figure 3. MDL-800 treatment does not influence HR repair.** (a) The schematic depictions of the HR efficiency assay. The HR reporter was linearized by I-SceI endonuclease *in vitro* to mimic DSBs. Then the purified linearized HR reporter (0.5 μg), along with 0.1 μg pCAG-DsRed vector were transfected into 2 × 10^5^ mouse iPSCs. FACS analysis was performed at 48-hour post transfection. (b) Analysis of HR efficiency in old murine-derived iPSCs treated with indicated doses of MDL-800 for 5 passages. The experiment was repeated three times. Error bars represent s.d., n.s. not significant.

**Supplementary figure 4. MDL-800 treatment does not cause alteration of cell cycle in old murine-derived iPSCs.** The old murine-derived iPSCs treated with MDL-800 for 5 passages were labelled with 10 μM EdU in a 5% CO_2_ and 3% O_2_ humidified incubator (Thermo Fisher Heracell 240i) at 37 °C for 2 hours. Then the cells were collected for fixation and permeabilization before the Click-iT reaction. Cells were also stained with Propidium iodide (PI) before FACS analysis. Error bars represent s.d.. The experiment was repeated for three times.

**Supplementary figure 5. MDL-800 promotes NHEJ and BER efficiency in a *Sirt6*-dependent manner.** Analysis of DNA repair efficiency in *Sirt6*^+/+^ and *Sirt6*^-/-^ mouse iPSCs treated with 20 μΜ MDL-800 for 5 passages. All experiments were repeated at least three times. Error bars represent s.d., * *P* < 0.05, *** *P* < 0.001, n.s. not significant.

**Supplementary figure 6. MDL-800 promotes formation of teratomas with all three germ layers from old murine-derived iPSCs.** Old murine-derived iPSCs were pretreated with MDL-800 for 5 passages before subcutaneously injected into groins of immunodeficient nude mice. (a) Representative images of teratomas. Scale bar: 5 mm. (b) Representative HE staining images showed that teratomas in MDL-800 treated group include tissues that originate from all three germ layers, while mesodermal tissues were absent in control group.

**Supplementary figure 7. MDL-800 promotes chimera formation from mouse iPSCs in a *Sirt6*-dependent manner.** The GFP tagged *Sirt6*^+/+^ or *Sirt6*^-/-^ mouse iPSCs were treated with 20 μΜ MDL-800 for 5 passages before blastocyst microinjection. Representative fluorescent images of E14.5 chimeric mouse embryos are shown in (a) and the percentage of GFP positive cells in E14.5 embryos is shown in (b). At least 25 embryos were included for each group. Scale bar: 2 mm. Error bars represent s.e.m., * *P* < 0.05, ** *P* < 0.01, n.s. not significant.
